# Nanocarriers: A novel strategy for the delivery of CRISPR/Cas systems

**DOI:** 10.3389/fchem.2022.957572

**Published:** 2022-07-26

**Authors:** Faranak Hejabi, Mohammad Sadegh Abbaszadeh, Shirinsadat Taji, Andrew O’Neill, Fatemeh Farjadian, Mohammad Doroudian

**Affiliations:** ^1^ Department of Cell and Molecular Sciences, Faculty of Biological Sciences, Kharazmi University, Tehran, Iran; ^2^ College of Art and Science, Syracuse University, Syracuse, NY, United States; ^3^ Department of Nanobiotechnology, Faculty of Biological Sciences, Tarbiat Modares University, Tehran, Iran; ^4^ Department of Clinical Medicine, Tallaght University Hospital and Trinity College Dublin, Dublin, Ireland; ^5^ Pharmaceutical Sciences Research Center, School of Pharmacy, Shiraz University of Medical Sciences, Shiraz, Iran

**Keywords:** nanocarriers, CRISPR/Cas9, gene therapy, nanomedicine, non-viral vector

## Abstract

In recent decades, clustered regularly interspaced short palindromic repeat/CRISPR-associated protein (CRISPR/Cas) has become one of the most promising genome-editing tools for therapeutic purposes in biomedical and medical applications. Although the CRISPR/Cas system has truly revolutionized the era of genome editing, the safe and effective delivery of CRISPR/Cas systems represents a substantial challenge that must be tackled to enable the next generation of genetic therapies. In addition, there are some challenges in the *in vivo* delivery to the targeted cells/tissues. Nanotechnology-based drug delivery systems can be employed to overcome this issue. This review discusses different types and forms of CRISPR/Cas systems and the current CRISPR/Cas delivery systems, including non-viral carriers such as liposomes, polymeric, and gold particles. The focus then turns to the viral nanocarriers which have been recently used as a nanocarrier for CRISPR/Cas delivery.

## 1 Introduction

**FIGURE 1:**
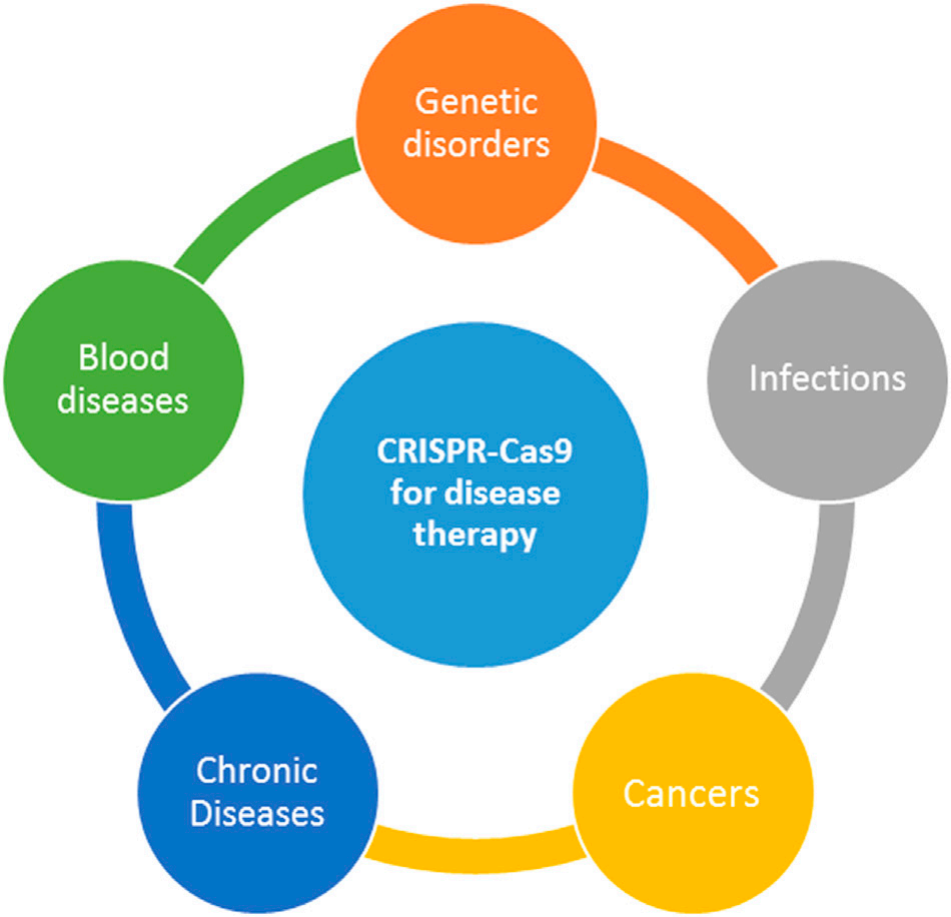
The CRISPR/Cas9 system has a wide diversity of applications for the treatment of different diseases.

Genome-editing technologies are crucial in the field of genetic engineering primarily due to the development of tools that make the manipulation of genetic material possible (Smyth, 2022; Nambiar et al., 2022). Although numerous genome-editing methods have been employed, the CRISPR-Cas approach has opened a new era for genetic modification as a result of its notable simplicity and versatility (Liu et al., 2022). The CRISPR/Cas system was first discovered in 1987 (Ishino et al., 1987) and was later found to be present in approximately 90% of archaea and 40% of bacteria. In 2005, the CRISPR/Cas system was revealed to be a component of the defensive system of prokaryotic cells against phages. In a landmark 2012 study, CRISPR/Cas systems were employed for gene-editing purposes (Jinek et al., 2012). Compared with other similar genome-editing tools, the CRISPR/Cas system has notable benefits including low cost and the fact that the system is relatively easy to use (Yang et al., 2022). In addition, the CRISPR/Cas system can overcome current genome-editing limits of gene overexpression or gene shut-down. Furthermore, CRISPR/Cas systems have been used successfully in a wide range of disease indications including bacterial infections, viral infections, and also cancers (Figure 1) (Chen et al., 2019; Uddin et al., 2020). As mentioned previously, the CRISPR/Cas system is part of the adaptive immune system of bacteria and archaea, playing a key role in the defensive immune response to a variety of stimuli including bacteriophages and mobile genetic elements (MGEs) by cleaving their genetic material. CRISPR/Cas systems are highly diverse across organisms and can be exchanged via horizontal gene transfer (Hasanzadeh et al., 2022). The CRISPR system is composed of two parts: single-guide RNA (sgRNA) and an endonuclease protein known as CRISPR-associated protein (Cas). The sgRNA identifies a precise genomic target sequence and leads the Cas nuclease to precisely cleave the detected DNA sequence. Induced double-stranded breaks are repaired through non-homologous end joining to generate insertions, deletions, or homology-directed repair for gene modifications (Petassi et al., 2020; Vo et al., 2021). Delivery of the CRISPR/Cas system is an attractive strategy because it is the most direct and rapid method for gene-editing purposes and has been shown to have fewer off-target effects and lower immunogenicity than other similar technologies (Wei et al., 2020). There still exist numerous challenges with the technology, with the development of improved delivery systems to enable effective systemic delivery of the CRISPR/Cas system a key challenge. Nanotechnology is a multidisciplinary field that has been increasingly capitalized for a variety of applications (Doroudian et al., 2019). Reducing the administered dose enhances the efficiency of drugs or active substances by specifically generating a synergistic effect and utilizing the multivalency of nanomaterial (Aghasadeghi et al., 2013; Doroudian et al., 2020). Furthermore, nanotechnology can also convert traditional delivery systems into smart carriers that are not only specifically transported to targeted tissues or cells, but also sustainably release payload in response to the microenvironmental stimuli like pH and temperature at targeted sites (Doroudian et al., 2021a). Protecting cargo against degradation by digestive elements is of significant importance for nucleic structures as they are highly exposed to the nucleases once entering the body. Therefore, nano-based delivery systems provide a solution to a number of key challenges in the context of therapeutic delivery (Doroudian et al., 2021b).

## 2 Types and classes CRISPR/Cas systems

**FIGURE 2:**
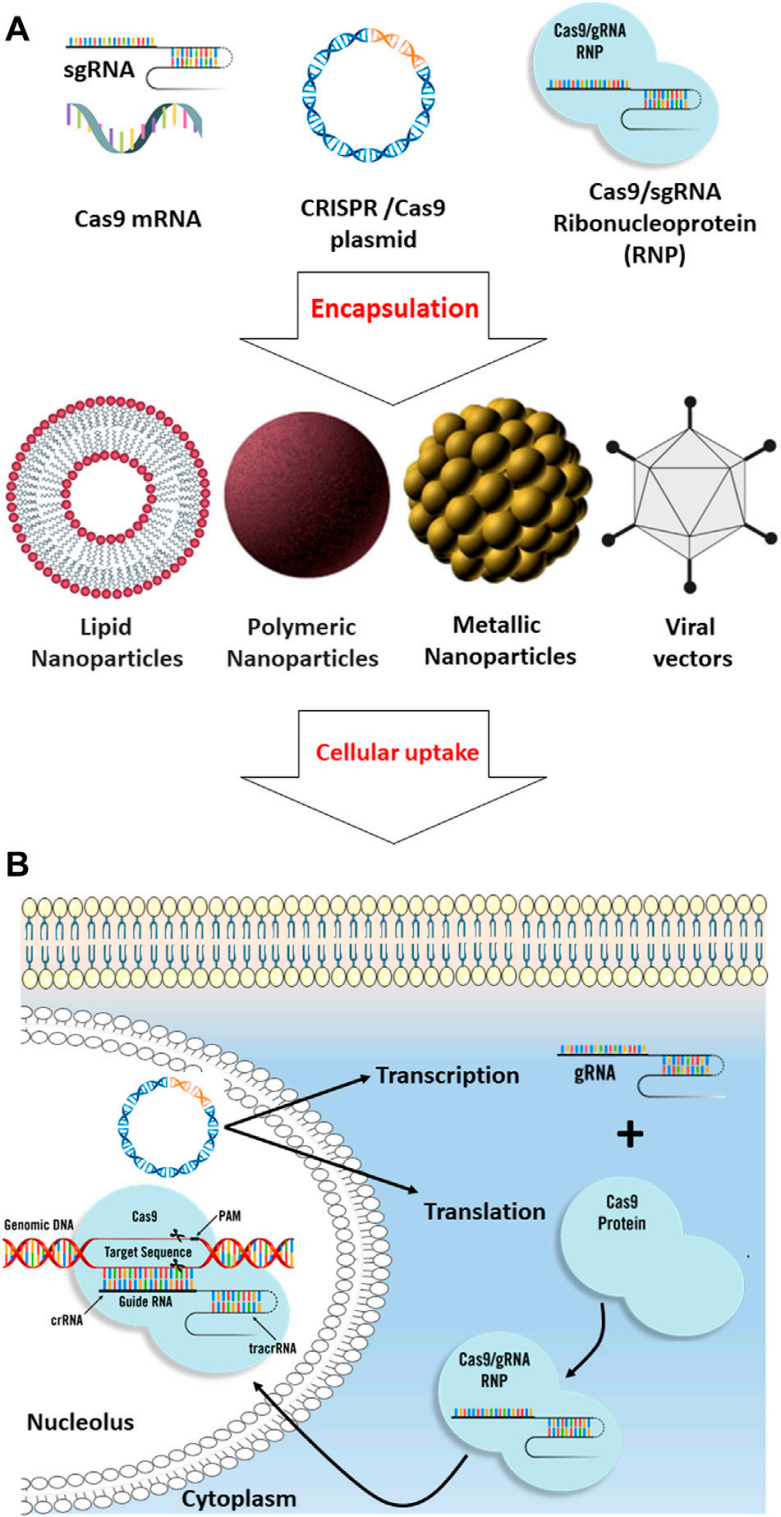
Comparison of different biomolecular CRISPR/Cas9 formats. **(A)** CRISPR/Cas9 systems include Cas9 plasmid, Cas9 mRNA, and sgRNA, and Cas9/sgRNA ribonucleoprotein can be encapsulated into different types of nanoparticles such as lipid-based, polymeric, metal-based, and viral nanodrug delivery systems for efficient intracellular uptake. **(B)** After cellular uptake through endocytosis, the CRISPR/Cas9 systems are released from the nanoparticles. For plasmid DNA delivery, the plasmid should be delivered into the nucleus, where the transcription mechanism must be employed to transcribe the gene into gRNA and Cas9 mRNA; in the cytoplasm, the Cas9 mRNA will be translated into the Cas9, and then, the gRNA and Cas9 protein will be transported back into the nucleus where the CRISPR mechanism can apply its effect on the targeted genomic DNA. For the Cas9 mRNA delivery strategy, the cargo should be released in the cytosol to allow the mRNA translation to Cas9 protein. Ribonucleoprotein (RNP) delivery is instantaneous compared to other strategies since the translation and transcription processes can be skipped, resulting in the immediate onset of gene editing.

Different types of CRISPR have been developed in recent years which are divided into various groups based on their functions and structures (Figure 2) (Nidhi et al., 2021). Sequence-similarity–based clustering and phylogenetic analysis of conserved Cas proteins are the main factors for the group arrangement/classification (Pinilla-Redondo et al., 2021). There are two main classes of CRISPR/Cas systems with each of them consisting of further subclasses. Class 1 consists of types I, III, and IV, while class 2 includes types II, V, and VI types (Makarova et al., 2020; Mohanraju et al., 2022). Class 1 systems are found in bacteria and archaea (Birkeland et al., 2017), while class 2 are only detected in bacteria that are not hyperthermophiles. The structure of proteins in class 2 systems is also less complex than those found in class I systems (Shmakov et al., 2017; Makarova et al., 2020). Different Cas proteins have different roles in the CRISPR‐Cas system. The Cas1 protein is a well-known integrase enzyme required for the specific breaking of a CRISPR array to insert a newly identified spacer in prokaryotic DNA (Givens et al., 2018; Roma-Rodrigues et al., 2020). The role of the Cas2 protein has not been identified thus far. However, this protein has RNase and DNase activities and is essential for the adaptation phase in *Escherichia coli* (*E. coli*) (Guzmán et al., 2021). The most frequently used subtype of CRISPR/Cas systems is the type II CRISPR/Cas9, employed for gene editing in eukaryotic cells (Table 1) (Makarova et al., 2020; Ramachandran et al., 2020).

**TABLE 1:** Classification and functions of different types of CRISPR.

Class	Type	Protein	Target	Function
Class 1	I	Cas (3,1,8)	DNA	Nuclease and helicase activity
Class 1	III	Cas (1,2,5,6,7,10)	DNA/RNA	Cleaving
Class 1	IV	Cas (5,7) and Csf1	Unknown	Cleaving
Class 2	II	Cas (1,2,4,9)	DNA	Cleaving
Class 2	V	Cpf1	DNA	Cleaving with cpf1

### 2.1 CRISPR/Cas9 forms

There are various formats for the CRISPR/Cas9 system, including 1) ribonucleoprotein complexes containing Cas9 endonuclease protein and sgRNA molecule, 2) a plasmid that encodes both Cas9 and sgRNA, 3) two separate plasmids each encoding Cas9 and sgRNA, and 4) Cas9 mRNA and sgRNA molecule (Table 2) (Deng et al., 2019; Cheng et al., 2021).

**TABLE 2:** CRISPR/Cas9 forms for disease therapy.

Format	Advantages	Disadvantages	Disease type
Plasmid DNA	• Cost-effective	• Longer lag time before Cas9 expression	Cystic fibrosis DMD
		• More prolonged determination of transgene product	
	• High stability	• High-risk off-target effects	
		• Nuclear entry of DNA	
mRNA	• No insertional mutagenesis	• Poor stability	Cataracts
	• Rapid expression of CRISPR components	• High cost of production	
Ribonucleoprotein (RNP)	• High editing effectiveness	• High cost	Fragile X syndrome DMD
	• Low risk of off-target effects		
	• Swift onset		
	• Low immunogenicity	• Bacterial endotoxin contamination	
	• No insertional mutagenesis		

#### 2.1.1 Plasmid form

Using a plasmid is a more stable and cost-effective mechanism in comparison to Cas9 mRNA and protein. The use of a plasmid form also further extends the production of Cas9 nuclease for constant gene editing (Ahmadi Danesh et al., 2012; Eoh and Gu, 2019). One of the advantages of the plasmid format is its ability to pass through cellular and nuclear membranes which is essential for the expression of the Cas9 nuclease protein in eukaryotic cells (Zhang et al., 2022). Therefore, the risk of off-target effects, random integration of the plasmid into the genome, and host immunologic responses may increase the continuous expression of plasmid in mammalian cells (Chen et al., 2020; Yang et al., 2021).

#### 2.1.2 mRNA form

Cas9 mRNA has attractive features such as effective nuclear entry, fast onset of gene editing, and the removal of the need for DNA transcription to mRNA. Moreover, encoding Cas9 in mRNA, which is translated in the cytoplasm, results in rapid protein expression and reduced off-target effects and insertion into the genome (Salehi Chaleshtori et al., 2012; Doroudian et al., 2013).

#### 2.1.3 Ribonucleoprotein form

The least complex approach is to transfer Cas protein with sgRNA specifically. This strategy offers the only temporal expression time and allows fast gene editing which removes the requirement for mRNA transcription and translation (Zhang et al., 2021; Yan et al., 2022). Furthermore, using the ribonucleoprotein strategy offers safe delivery to protect endonuclease Cas9 and protect RNA from rapid degradation. However, some challenges must be considered such as low efficiency for cellular uptake, high risk of endotoxin contamination, and high immunogenicity (Chen et al., 2020; Yang et al., 2021).

## 3 Non-viral nano delivery systems

Biomacromolecular therapeutic agents, including DNAs and RNAs, are often degraded in biological fluids (Doroudian et al., 2021b; Azhdari et al., 2022). Moreover, they are ineffective at crossing the cell‐membrane barrier due to their hydrophilicity and negative charge (Hanjani et al., 2022). Therefore, nanocarriers are frequently applied to overcome these limitations and these are frequently used to deliver CRISPR/Cas9 to target cells (Wan et al., 2021). Nano delivery systems enhance the stability of biomacromolecular therapeutic agents and protect them from premature degradation and rapid clearance *in vivo*, thereby enabling the delivery of their drug content to the target area (Eoh and Gu, 2019; Roma-Rodrigues et al., 2020; Kazemian et al., 2022). Nano delivery systems present a promising strategy for overcoming current challenges with CRISPR/Cas9 delivery. In this context, nanoparticles are used as carriers for the direct delivery of therapeutic components to the nuclei of targeted cells (Wang et al., 2017; Doroudian et al., 2021b). Nano delivery systems improve gene delivery by covering the negative charge of DNA, therefore preventing the cargo from intracellular nuclease degradation (Li et al., 2022). This is achieved by pressing DNA through electrostatic interactions between anionic DNA and polycations, encapsulating it with biodegradable polymers, or adsorbing it (Ibraheem et al., 2014; Doroudian et al., 2017). Chemical transfection transfers cargo into target cells using chemical vectors such as lipid vesicles and polymer-based chemicals. The chemical vectors work as a carrier system for encapsulating genetic-editing plasmid DNA and mRNA (Duan et al., 2021; Han et al., 2022; Mahmoud et al., 2022). Inorganic formats of nucleic acid delivery, such as gold nanoparticles, carbon nanotubes, and graphene, also produce significant performance. Protein and other biomolecules can also be delivered using chemical transfection (He et al., 2020; Hosseinkazemi et al., 2022). Polymeric carriers are chemically diverse and versatile. Polyethylenimine (PEI), chitosan, and poly (l-lysine) have been the most common polymeric DNA vectors (PLL) (Wang et al., 2017; Ashok et al., 2021).

### 3.1 Lipid nanoparticles

Liposomes are promising nanodrug delivery systems owing to their similarity to the plasma membrane. Lipid-based nanoparticles are one of the most widely used nanoparticles for nucleic acid delivery (Filipczak et al., 2020). The lipid layer enhanced the ability of the CRISPR cargo to cross the cell membrane and also to preserve it from RNases, enzymatic degradation, and immune responses (Liu et al., 2018a; Liu et al., 2018b). Furthermore, Lipofectamine, RNAiMAX, TurboFectTM, and StemfectTM are commercially available lipid-based nanoparticles that have been employed for CRISPR plasmids, mRNA, gRNA, and Cas9-sgRNA RNP delivery (Mout et al., 2017; Givens et al., 2018; Chen et al., 2020). Liposomes have been shown to be promising carriers for CRISPR/Cas9 delivery. The lipid bilayer protects protein and nucleic acid from decomposition in the blood circulatory system (Qiu et al., 2021). It can also increase endosomal escape via fusing with the endosomal membrane (Quagliarini et al., 2021). It has been shown that CRISPR/sgRNA RNPs carried by cationic liposome could detect 65% of reporter gene disruption in U2OS EGFP cells, a human osteosarcoma cell line. It was also identified that Cas9/sgRNA RNP delivery via cationic liposomes results in an approximately 13% degradation of the reporter gene near the injection site of the inner ear in a murine *in vivo* study. Furthermore, it has been shown that cationic liposomes can be an effective vehicle for genome-editing platform delivery (Zuris et al., 2015). A temporary cytosolic delivery system for correct Cas9 ribonucleoprotein is critical in the CRISPR/Cas9 target specificity. The improvement of the delivery system remains the main challenge due to the large size of the Cas9 protein and a negative-strand RNA (Hou et al., 2021). To address this issue, a novel nanocarrier was employed, which was a size-controlled lipopeptide-based nanosome structure derived from the blood–brain barrier–permeable dNP2 peptide to deliver a hyper-accurate Cas9 RNP complex (HypaRNP) into human cells for gene editing (Lin et al., 2022). The use of encapsulated HypaRNP in an *in vivo* model resulted in rapid nuclear localization without causing cytotoxicity. Thus, the HypaRNP effectively silenced endogenous eGFP in human embryonic kidney cells and glioblastoma cells (Saadati and Hosseini, 2012; Thach et al., 2019). This strategy was also used to modify the dipeptidyl peptidase-4 gene (DPP-4) to upregulate the glucagon-like peptide 1 in a murine model of type 2 diabetes mellitus (T2DM) db/db mice. The nanocarrier, loaded with the Cas9 ribonucleoprotein complex effectively blocked the expression of the DPP-4 gene in T2DM mice. Normalized blood glucose levels followed the decrease in DPP-4 enzyme activity and improved insulin response with less liver and kidney damage. These effects were similar to those of sitagliptin, a new chemical DPP-4 inhibitor that requires repeated dosing. This demonstrates that a nano-liposomal carrier system containing therapeutic Cas9 ribonucleoprotein has enormous potential as a tool for improving gene therapy for human liver disorders (Cho et al., 2019). To deliver Cas9 mRNA and sgRNA, a liposome (LNP-INT01) was developed with biodegradable ionizable lipid, and PEG-DMG. LNP-INT01 half-life cycle in the liver was just around 6 h. As a result, it was safer than other forms of non-degradable liposomes used in treatment. LNP-INT01 consisting of Cas9 mRNA and sgRNA was supplied to CD-1 mice at various doses to downregulate serum TTR levels. Using a single dose at the peak of three mpk, serum TTR levels were reduced by more than 97 percent for more than 12 months compared to controls. Furthermore, it was confirmed that the majority of the LNP-INT01 was accumulated in the liver. Thus, approximately 70% of the resulting DNA editing was found in the liver. Furthermore, the spleen and kidney were shown to have very poor levels of editing, indicating organ specificity. As a result, this type of LNP-INT01 is intended to be used in clinical trials for liver-based genetic disorders (Table 3) (Finn et al., 2018).

**TABLE 3:** Examples of CRISPR/cas9 disease therapy based on clinical trials.

Disease type	Treatment	Phase	Year	National clinical trial (NCT) number
Sickle-cell disease	Genetic: GPH101 drug product	1/2	2021	NCT04819841
Sickle-cell disease	Drug: CRISPR_SCD001	1/2	2021	NCT04774536
Lymphoma	Drug: Cyclophosphamide	1	2020	NCT04637763
Hereditary transthyretin amyloidosis	NTLA-2001	1	2020	NCT04601051
Viral keratitis	Drug: BD111 adult single-group dose	1/2	2020	NCT04560790
B acute lymphoblastic leukemia	Drug: PBLTT52CAR19	1	2020	NCT04557436
Enterovirus infections	Non-invasive detection method: CRISPR technology	_	2020	NCT04535648
Renal cell carcinoma	CTX130	1	2020	NCT04438083
Relapsed or refractory multiple myeloma	CTX120	1	2020	NCT04244656
Gastro-intestinal cancer	Drug: CTX/Fludarabine/IL-2 TIL	1/2	2020	NCT04426669
Kabuki syndrome 1	Intervention on primary cultured cells	_	2019	NCT03855631
B-cell malignancy	CTX110	1/2	2019	NCT04035434
Relapsed or refractory CD19^+^ leukemia or lymphoma	Genetic: XYF19 CAR-T cells Drug: CTX/Fludarabine	1	2019	NCT04037566

### 3.2 Polymeric nanoparticles

Polymers are extensively used in drug delivery applications (Kanduri et al., 2021; Lin et al., 2022). The homology-independent targeted integration (HITI) approach, which has demonstrated improved site-specific transgene integration frequencies, facilitates successful CRISPR/Cas9-mediated knock-in of therapeutic genes in non-dividing cells *in vivo*. Supramolecular nanoparticle (SMNP) carriers were used in a study to deliver two DNA plasmids, the CRISPR/Cas9 genome-editing framework and a therapeutic gene, Retinoschisin 1 (RS1), allowing CRISPR/Cas9 knock-in of the RS1 gene with HITI (Chou et al., 2020). In another study, Xiao-Yan He et al. demonstrated that efficient reversal of tumor immunosuppression is vital in cancer therapy. A nano delivery system for CRISPR/Cas9 plasmid was developed to reverse tumor immunosuppression by silencing the β-catenin gene. As a targeted nano delivery system, it was decorated with aptamer-conjugated hyaluronic acid and peptide-conjugated hyaluronic acid. This strategy resulted in a dramatically increased plasmid accumulation in malignant cell nuclei. The genome-editing system can effectively induce β-catenin knockout and inhibit the Wnt/β-catenin pathway, resulting in substantially downregulated proteins associated with tumor growth and immunosuppression (He et al., 2020). Cationic polymer polyethyleneimine-β-cyclodextrin (PC), which is proven helpful for small plasmid transfection, can efficiently deliver Cas9 and sgRNA plasmids. However, PC can compact and capture large plasmids with a high N/P ratio but plasmids show results in effective editing at two genome loci, namely, hemoglobin subunit beta (19.1%) and rhomboid five homologs 1 (RHBDF1) (7.0%). Furthermore, Sanger sequencing indicates that genome editing at these loci was effective (Zhang et al., 2019). In another study, Ami Jo and colleagues developed a nanoparticle carrier using poly lactic-co-glycolic acid (PLGA) to deliver a CRISPR–Cas9 plasmid into the bone marrow. In this system, the authors engineered a PLGA-based nano delivery system composed of several copies of plasmid which have been fluorescently labeled with TIPS pentacene fluorophore which were then encapsulated by the PLGA polymer. Following 24-h of treatment, most DNA including two to three copies of plasmid per nanoparticle were released and *in vitro* screening results demonstrated a majority of TIPS pentacene positive cells and the detection of Cas9 proteins inside the cells (Jo et al., 2020). Rohiwal et al. also attempted to characterize nanoparticles used to deliver plasmid DNA of the CRISPR/Cas system *in vitro* to eukaryotic cells. Magnetic nanoparticles (MNPs) were synthesized implementing CRISPR/Cas9-complexed polyethyleneimine (PEI). They evaluated effective homology-directed repair (HDR) and non-homologous end-joining (NHEJ) events following nanoparticle transfection using a robust HEK-293 cell line expressing the traffic light reporter (TLR-3) system. MNPs were generated through the co-precipitation process, with an average particle size of about 20 nm. The dynamic light scattering and zeta potential measurements revealed that the NPs had a small size range and adequate colloidal stability. This method investigated the use of non-viral delivery of CRISPR/Cas9 and a DNA template to execute HDR and NHEJ in the same assay. The results show that PEI-MNPs are an effective delivery vehicle for plasmids form of CRISPR/Cas9 and template DNA in comparison with the standard lipofectamine transfection. Moreover, this type of delivery system has the potential to increase the safety and efficiency of genetic modifications (Rohiwal et al., 2020). Further research has also documented a sequence of reducible branched ester-amine quadpolymers (rBEAQs) that were generated and tested for their ability to co-encapsulate and deliver DNA plasmids and RNA oligonucleotides. The goal of using these engineered rBEAQs was to use polymer branching, reducibility, and hydrophobicity to efficiently co-encapsulate DNA and RNA in nanoparticles at low polymer-to–nucleic acid w/w ratios while enabling high distribution efficiency. Moreover, they also evaluated the development of a safe DNA–siRNA co-delivery and also non-viral CRISPR-mediated gene editing using Cas9 DNA and sgRNA co-delivery (Rui et al., 2019).

### 3.3 Metallic nanoparticles

Gold is among the most commonly used metals in biomedicine as a result of its anti-inflammatory and antibiotic properties (Ko et al., 2022). In recent years, various applications of gold nanoparticles as vectors in therapeutic strategies have been investigated (Trigueros et al., 2019; He et al., 2021; Jahangiri-Manesh et al., 2022). Coelho et al. recently designed a drug delivery nanosystem based on pegylated gold nanoparticles with anthracycline and tyrosine kinase inhibitor doxorubicin, a small anti-cancer molecule, and varlitinib, an inhibitor of the tyrosine kinases, for a combination strategy against pancreatic cancer cells (Coelho et al., 2019). In a recent study, gold nanoparticles were used for combined gene and antimicrobial therapy by binding antimicrobial peptides with cationic gold nanoparticles for gene delivery to mesenchymal stem cells (Peng et al., 2016). While CRISPR/Cas-based fluorescent biosensors have been produced recently, they still need amplification for measurement. The first CRISPR/Cas12a-based nucleic acid amplification-free fluorescent biosensor has been newly produced by Jin-Ha Choi’s team to detect cfDNA through the metal-enhanced fluorescence phenomenon (MEF) using DNA-functionalized gold nanoparticles. Moreover, MEF occurred with color shifts from purple to red-purple after activating the CRISPR/Cas12a complex with the target cfDNA and corresponding single-strand DNA (ssDNA) degradation between gold nanoparticles and fluorophore. Breast cancer gene-1 (BRCA-1) can be diagnosed with high sensitivity in 30 min using this method. This fast and highly selective sensor can be used to quantify other nucleic acid biomarkers, including viral DNA, in a field-deployable and point-of-care testing (POCT) framework (Imani et al., 2011; Jahangiri-Manesh et al., 2022). Generally, the research by Rabiee and his collaborators emphasizes the role of various parameters in developing a non-viral vector in gene delivery carriers. Work is ongoing in the context of 2D crystals, precisely layered double hydroxides, because of their considerable adjustability and cost-effective preparation process. Hence, the relationship between various physicochemical properties parameters of LDH, such as pH, scale, zeta potential, and synthesis process, was evaluated and adjusted for CRISPR/Cas9 delivery and reverse fluorescence response to EGFP. ZnAl LDH and ZnAl HMTA LDH were produced, characterized, and used to deliver CRISPR/Cas9 to the HEK-293 cell line throughout this study. The analysis demonstrated adequate binding potential with the DNA, making it an effective vector for CRISPR/Cas9 delivery (Rabiee et al., 2020). Furthermore, another independent study showed the advantages of biomimetic cancer cell–coated zeolitic imidazolate frameworks (ZIFs) for cells-specific delivery of genome-editing systems. This study found that coating ZIF-8 with a cancer cell membrane and encapsulating CRISPR/Cas9 (CC-ZIF) resulted in evenly coated cell membrane–localized C3-ZIF. Furthermore, when C3-ZIFMCF was incubated with MCF-7, HeLa, HDFn, and aTC cell lines, MCF-7 cells presented the highest uptake, while non-cancerous cells showed minimal uptake. In terms of genome editing, MCF-7 was transfected with C3-ZIFMCF, which resulted in 3-fold repression of EGFP expression compared to 1-fold repression of EGFP expression when MCF-7 was transfected with C3-ZIFHELA. Alyami et al. described *in vivo* testing confirmed C3-ZIFMCF’s selectivity to aggregate in MCF-7 tumor cells (Alyami et al., 2020).

### 3.4 Viral vectors

Viral vectors have been developed over the last 30 years for gene therapy to deliver various nucleic acid-based therapeutics, and some have been approved for clinical use. Despite their safety concerns and the possibility of introducing undesired mutations, viral delivery systems have one of the most efficient strategies to deliver plasmid-based nucleic acids to mammalian cells *in vitro* and *in vivo* thus far (Bulcha et al., 2021). Adenovirus and lentivirus have been employed frequently among all other viruses (Zheng et al., 2018). Adeno-associated viruses (AAVs) are single-stranded DNA viruses that contain two genes (Berns and Giraud, 1996). Recombinant gene delivery vector systems (rAAVs) were first generated based on AAV serotype 2 in the 1980s. Currently, rAAVs are the leading platform for *in vivo* delivery of gene therapies (Duncan, 2022). Nano-sized viral delivery systems offer numerous advantages, including the highest level of biological safety with limited pathogenic properties. In addition, rAAV provides efficient gene delivery and sustained transgene expression in multiple tissues, including muscle, lung, liver, and central nervous system. Furthermore, rAAV has enjoyed some success in human clinical trials. AAV is the most popular viral vector to treat cancer due to its oncolytic therapy ability (Senís et al., 2014; Ibraheim et al., 2018; Lino et al., 2018; Wang et al., 2020). For example, *in vivo* as well as *ex vivo* genome editing on *KRAS*, *p53*, and *LKB1*, using AAV, lentivirus-, or particle-mediated delivery of guide RNA in neurons, immune cells, and endothelial cells has been successfully accomplished due to simultaneously modeled dynamics of the top three significantly mutated genes in lung adenocarcinoma (Platt et al., 2014). In the viral delivery method, the adenovirus is the most important and most commonly used due to its broad range of serotype specificity, non-pathogenicity, and less immunogenicity (Chew et al., 2016; Li et al., 2019; Yu and Wu, 2019; Wang et al., 2020). In 2012, the EU approved the first AAV-based gene therapy drug, Glybera, for patients with lipoprotein lipase deficiency, suggesting the great promise of AAV for gene therapy. Recombined rAAV can reduce the off-targeting rate due to its capability to identify the target cell more efficiently (Gaj et al., 2016; Bak and Porteus, 2017; Hanlon et al., 2019; Krooss et al., 2020). In the long term, it is important that we investigate further the integration of the CRISPR/Cas9 system in host DNA (Li et al., 2015; Heck and Brault, 2018; Uchida et al., 2021).

## 4 Conclusion

The CRISPR/Cas9 system is a practical, low cost, and highly efficient tool, recently used for genome editing in comparison with other genome-editing tools such as TALEN and ZFN. Although, choosing a suitable carrier for delivery is still challenging, the presence of a wide range of delivery materials is resulting in improved access. Furthermore, physical, chemical, and biological transfer methods have their own unique advantages and disadvantages. In this article, we have focused on these very advantages and disadvantages to highlight the recent advances in the field to provide a balanced outlook on current developments. As for future perspectives, one of the most challenging parts of genomic editing technology, particularly in clinical applications, is to develop active targeting nanodrug delivery systems. This strategy provides efficient and effective tools for the manipulation of certain cells/tissues. Smart nanodrug delivery systems, including targeted nanodrug carriers and stimuli-responsive nanoparticles, are promising novel approaches that can be employed to tackle these challenges. These novel strategies would provide a near-term future in which the therapy of genetic disorders is within our reach.
